# The application of mRNA-based gene transfer in mesenchymal stem cell-mediated cytotoxicity of glioma cells

**DOI:** 10.18632/oncotarget.10835

**Published:** 2016-07-25

**Authors:** Xing-Rong Guo, Zhuo-Shun Yang, Xiang-Jun Tang, Dan-Dan Zou, Hui Gui, Xiao-Li Wang, Shi-Nan Ma, Ya-Hong Yuan, Juan Fang, Bin Wang, Li Zhang, Xu-Yong Sun, Garth L. Warnock, Long-Jun Dai, Han-Jun Tu

**Affiliations:** ^1^ Hubei Key Laboratory of Stem Cell Research, Taihe Hospital, Hubei University of Medicine, Shiyan, China; ^2^ Department of Neurosurgery, Taihe Hospital, Hubei University of Medicine, Shiyan, China; ^3^ Department of Animal Care Center, Hubei University of Medicine, Shiyan, China; ^4^ The Biomedical Research Center, University of British Columbia, Vancouver, Canada; ^5^ Guangxi Key Laboratory for Transplantation Medicine, 303 Hospital of PLA, Nanning, China; ^6^ Department of Surgery, University of British Columbia, Vancouver, Canada

**Keywords:** glioblastoma, cancer therapy, mesenchymal stem cells, TRAIL, PTEN

## Abstract

Since the tumor-oriented homing capacity of mesenchymal stem cells (MSCs) was discovered, MSCs have attracted great interest in the research field of cancer therapy mainly focused on their use as carries for anticancer agents. Differing from DNA-based vectors, the use of mRNA-based antituor gene delivery benefits from readily transfection and mutagenesis-free. However, it is essential to verify if mRNA transfection interferes with MSCs' tropism and their antitumor properties. *TRAIL*- and *PTEN*-mRNAs were synthesized and studied in an *in vitro* model of MSC-mediated indirect co-culture with DBTRG human glioma cells. The expression of TRAIL and PTEN in transfected MSCs was verified by immunoblotting analysis, and the migration ability of MSCs after anticancer gene transfection was demonstrated using transwell co-cultures. The viability of DBTRG cells was determined with bioluminescence, live/dead staining and real time cell analyzer. An *in vivo* model of DBTRG cell-derived xenografted tumors was used to verify the antitumor effects of *TRAIL*- and *PTEN*-engineered MSCs. With regard to the effect of mRNA transfection on MSCs' migration toward glioma cells, an enhanced migration rate was observed with MSCs transfected with all tested mRNAs compared to non-transfected MSCs (*p*<0.05). *TRAIL*- and *PTEN*-mRNA-induced cytotoxicity of DBTRG glioma cells was proportionally correlated with the ratio of conditioned medium from transfected MSCs. A synergistic action of TRAIL and PTEN was demonstrated in the current co-culture model. The immunoblotting analysis revealed the apoptotic nature of the cells death in the present study. The growth of the xenografted tumors was significantly inhibited by the application of MSC_PTEN_ or MSC_TRAIL/PTEN_ on day 14 and MSC_TRAIL_ on day 28 (*p*<0.05). The results suggested that anticancer gene-bearing mRNAs synthesized *in vitro* are capable of being applied for MSC-mediated anticancer modality. This study provides an experimental base for further clinical anticancer studies using synthesized mRNAs.

## INTRODUCTION

Mesenchymal stem cell (MSC)-mediated strategy holds great potential for the development of patient-tailored antitumor therapy [1–3]. It is able to produce specific anticancer agents locally and constantly. As a direct attacker, an anticancer gene that was pre-engineered on MSCs plays a critical role for the action in this system. A number of vectors have been widely used in gene therapy-related studies, including four major types: plasmids, viral vectors, cosmids and artificial chromosomes. Common to all engineered vectors are an origin of replication, a multicloning site, and a selectable marker. Current viral vectors for gene therapy are associated with serious safety concerns [4, 5] and nonviral DNA-based vectors are limited by low transfection efficiency and the potential of insertional mutagenesis [6]. In recent years, novel stabilized mRNA constructs have become more attractive alternatives to the most commonly used DNA-based plasmid (pDNA) [7].

The use of mRNA as a carrier for therapeutic genes in gene therapy has been mainly attributed to the discovery of 5′mRNA anti-reverse cap analogues (ARCA), the insertion of additional untranslated regions, and poly(A) tails that significantly enhance efficient translation of foreign mRNA in host cells [7–10]. In general, mRNA-based gene delivery has considerable advantages compared to pDNA delivery in gene therapy applications: (i) mRNA is translated in the cytoplasm directly and does not require transfer into the nucleus. Therefore, the mRNA transfection is efficient even when host cells are no longer dividing, which is a major drawback of pDNA transfection; (ii) because of the nature of RNA, the risk of insertional mutagenesis is apparently omitted; (iii) Toll-like receptor-activated immunogenic response to mRNA is much less pronounced compared to unmethylated CpG motifs of DNA recognized by TLR9; and (iv) the mRNA construct is far smaller than pDNA and is presumably transfected into host cells easily [7, 11]. MSC-mediated antitumor modality requires tumor-specific antituor gene(s) to be engineered into MSCs prior to transplanting them into the patient with cancer [3].

The prerequisites for MSC-mediated antitumor strategy include efficient gene transfection and adequate gene expression in MSCs. DNA-based vectors were used in almost all previous work including ours in this field of cancer research, such as IFN-α (adenoviral vector [12]), IFN-β (adenoviral vector [13]), INF-γ (adenoviral vector [14]), IL2 (adenoviral vector [15]), IL12 (adenoviral vector [16]), TRAIL (lentiviral vector [17, 18], plasmid vector [19–21]) and PTEN (plasmid vector [3]). During the recent few years, the application of mRNA-based vectors has become more attractive especially in the research field of iPSC [22–25]. In the present study, we initiated the application of this advantageous tool in the field of cancer research. The cytotoxic effects of *TRAIL* (TNF-related apoptosis-inducing ligand) and *PTEN* (phosphatase and tensin homolog) engineered MSCs through mRNA vectors on malignant glioma cells were determined *in vitro*, and hopefully, this study could contribute to the exploration of clinically meaningful therapeutic strategies for cancer patients.

## RESULTS

### Characteristics of MSCs and DBTRG cells

**Figure 1:**
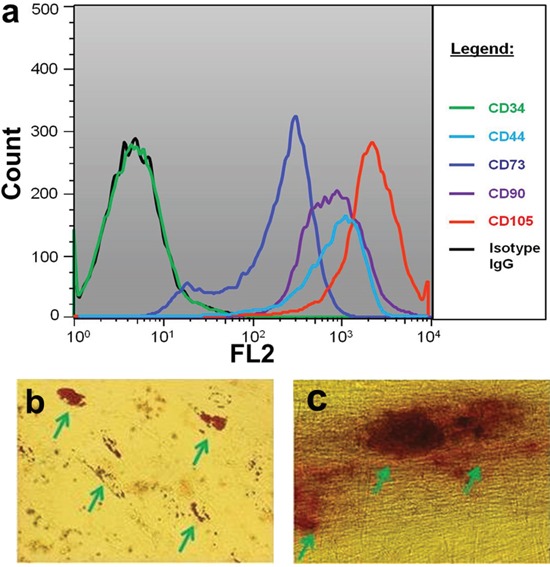
Characteristics of MSCs **a.** Biomerker characterization using flow cytometry; **b.** Adipogenic differentiation of MSCs stained with Oil Red O; **c.** Osteogenic differentiation of MSCs stained with Alizarian Red. Green arrows pointed positive cells.

The MSCs used in the present study exhibited positive expression of CD44^+^, CD73^+^, CD95^+^, CD105^+^ and negative of CD34^−^ (Figure 1a). These cells differentiated to adipogenic and osteogenic cells under specific differentiation conditions (Figure 1b and 1c). According to the criteria established by ISCT [26], the characterization of membrane biomarker and the capacity of functional differentiation meet the standards of human MSCs. Normal karyotype (46 _XY_) was observed in MSCs which was consistent with bone marrow-derived MSCs in our previous study [27]. However, DBTRG cells were demonstrated abnormal chromosome karyotype (84_XX_).

### TRAIL- and PTEN-bearing mRNA construction and their expression in MSCs

**Figure 2:**
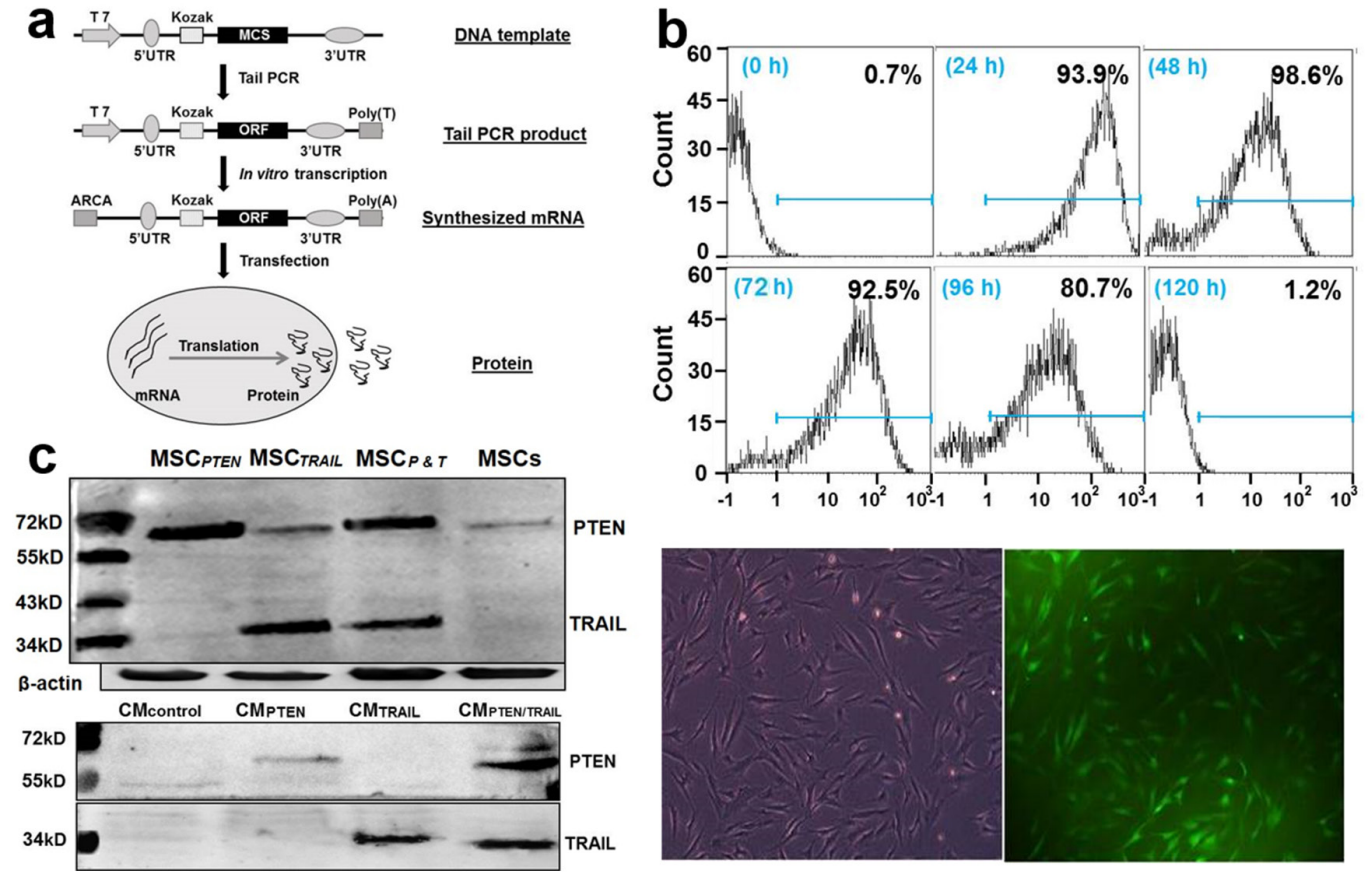
mRNA synthesis and *in vitro* expression in MSCs **a.** Flow chart of mRNA synthesis *in vitro*; **b.** Assessment of transfection efficiency in *GFP*-mRNA-transfected MSCs. Top panel shows the FACS results of time-dependent changes of GFP-positive MSCs after *GFP*-mRNA transfection; Lower panel displays a representative microscopic field under regular light (left) and green fluorescent light (right) respectively. The images were taken from MSC culture 2 days after transfection. The original magnification, 400X; **c.** Immunoblotting analysis of TRAIL and PTEN in cell lysates (top panel) and conditioned media (bottom panel).

Antitumor gene-bearing mRNAs were synthesized as illustrated in Figure 2a. In order to make both TRAIL and PTEN product secretory, a leading sequence corresponded to an 18 amino acid segment (MKFPSQLLLLLLFGIPGM) was introduced into the DNA template. Transacting activator of transcription (TAT, YGRKKRRQRRR) was inserted into *PTEN* template for its transmembrane purpose. The DNA sequence was verified by restriction enzyme digestion and sequencing analysis (data not shown). The transfection efficiency was tested using a synthesized *GFP*-mRNA in MSCs. A very high transfection efficiency was observed using fluorescence microscopy (lower panel in Figure 2b). The FACS results of time-dependent changes of GFP-positive MSCs indicated that the transfected *GFP*-mRNA retained its translational activity up to 4 days after transfection (top panel in Figure 2b). As shown in Figure 2c, the expression of TRAIL and PTEN was clearly enhanced in transfected MSCs. The endogenous PTEN was observed in control MSCs with slightly different size, whereas, the expression of endogenous TRAIL was undetectable under this experimental condition. The secreting forms of PTEN and TRAIL were also detectable in the corresponding CMs (Figure 2c).

### The effects of mRNA transfection on MSC migration

**Figure 3:**
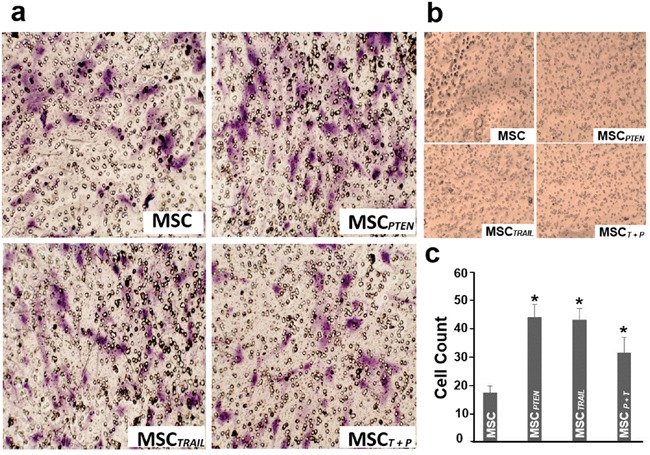
*In vitro* migratory ability of MSCs **a.** The migratory ability of native MSCs, MSC*_TRAIL_*, MSC*_PTEN_* and MSC*_TRAIL/PTEN_* (MSC*_T+P_*) toward DBTRG glioma cells using a transwell co-culture system. Representative photomicrographs of stained membrane show migrated cells. Magnification, 100x. **b.** MSCs’ migration test using native MSCs as control cells at the bottom of the chamber. **c.** Summary of three independent experiments was presented as mean ± SEM. The cell number of each filter membrane was obtained from the average of five randomly selected microscopic fields. * *p* < 0.05 *vs.* native MSC.

MSC migration was determined by transwell system. After 48 hrs of co-culture, a considerable number of cells (*i.e.* native MSC, MSC*_TRAIL_*, MSC*_PTEN_* and MSC*_TRAIL/PTEN_*) migrated across the microporous membrane toward DBTRG cells (Figure 3a), while there was no cell migrated toward normal MSC control cells (Figure 3b). Compared with control MSCs, the transfection of *TRAIL*- and *PTEN*-mRNAs did not obstruct MSC's migration toward DBTRG glioma cells (Figure 3c). Interestingly, the MSC migration was significantly enhanced by single gene transfection (MSC*_TRAIL_* or MSC*_PTEN_*) or double gene transfection (MSC*_TRAIL/PTEN_*).

### The effects of *TRAIL*- and *PTEN*-engineered MSCs on the viability of DBTRG glioma cells

**Figure 4:**
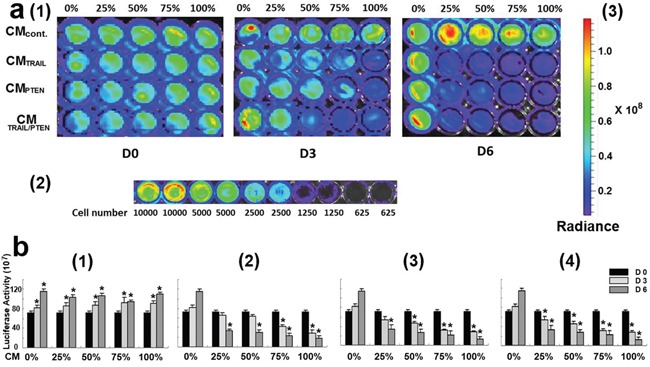
a. Assessment of DBTRG cell viability using bioluminescence determination (a1) Representative measurement of luminescence intensity. Cell culture medium was indicated on the left side of the graph. CM ratios and time points were labeled at the top and bottom respectively. The luminescence intensity of each well was determined by IVIS Spectrum System 10 min after adding D-luciferin. (a2) Sensitivity test of IVIS Spectrum System. The bioluminescence signal was not detectable when the cell number was less than 625 cells/well. (a3) Luminescence scale. Color scale: Min = 6.31 × 10^6^; Max = 1.19 × 10^8^. Radiance intensity was expressed as p/sec/cm^2^/sr. **b.** Summary of DBTRG cell viability. DBTRG cells were co-cultured with CM_control_ (b1), CM_TRAIL_ (b2), CM_PTEN_ (b3) and CM_TRAIL/PTEN_ (b4). The relative cell viability was represented as luciferase activity. Data were presented as mean ± SEM. ^*^ *p* < 0.05, compared with control (day 0) at the same CM ratio in b1 and compared with control (0%) at the same time point in b2-b4.

**Figure 5:**
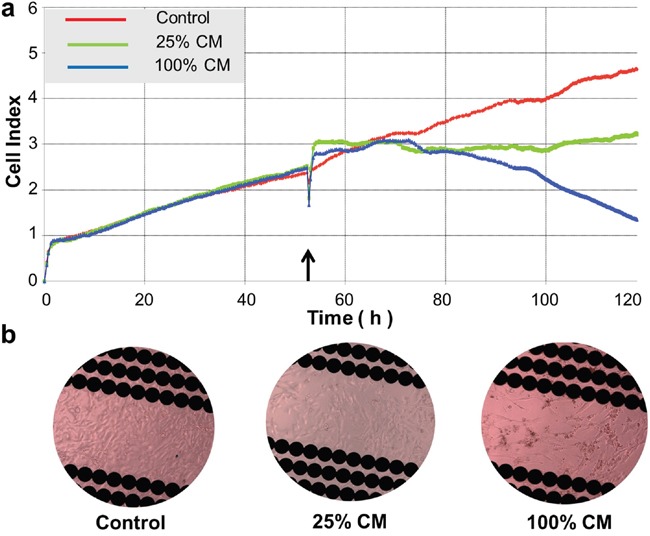
Real-time assessment of conditioned medium (CM)-induced cytotoxicity in DBTRG cells **a.** Real-time monitoring of CM-induced cytotoxicity in DBTRG cells. Cell index was automatically recorded with the xCELLigence real-time cell analyzer (RTCA) every 5 min until the end of the experiment (120 h). Each tracing represents an average of three parallel assessments. The arrow indicates the time when the culture medium was replaced with CM_PTEN_ with different ratios. **b.** Microscopic observation of BBTRG cells in the E-Plate. Images were taken from the E-Plate 16 of the xCELLigence at the end of the experiment and representative image was shown from each setting. Original magnification, 400x.

The indirect co-culture was used to determine the effects of *TRAIL*- and *PTEN*-engineered MSCs on the viability of DBTRG glioma cells using luminescence, real-time cell analyzer (RTCA) and fluorescence microscopy. As shown in Figure 4a, the intensity of luminescence in DBTRG cells decreased with the increase of incubation time and CM ratio. All test results were summarized in Figure 4b. Compared with control (day 0) at the same CM_CONTROL_ with various ratios, the cell viability was significantly increased with incubation time in all tested ratios (b1, *p* < 0.05). As shown in b2-b4, starting at low CM ratio (25%), all cells incubated with CM_TRAIL_, CM_PTEN_ or CM_TRAIL/PTEN_ revealed significant cell death (*p* < 0.05) at day 6. At day 3 however, the significant cell death (*p* < 0.05) started to appear at CM ratio 75% for CM_TRAIL_, 50% for CM_PTEN_ and 25% for CM_TRAIL/PTEN_. However, RTCA results indicate that CM_PTEN_-induced changes of cell viability started at about 20 h after CM treatment (Figure 5).

**Figure 6:**
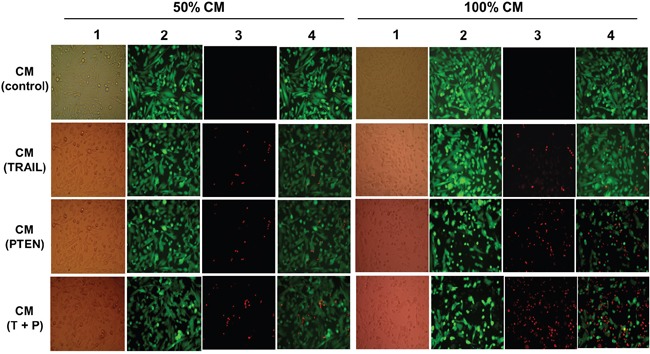
DBTRG cell viability of indirect co-cultures DBTRG cells were incubated in various CMs (indicated on the left side of the graph) at different ratios (indicated on the top). LIVE/DEAD staining was performed on day 4 after initiation of the indirect co-culture. Column 1 (brightfield): whole population of cells which still attached to the culture surface; column 2: live cells stained with calcein are green; column 3: dead cells stained with EthD-1 show red; column 4: merged images. Original magnification, 400x.

**Figure 7:**
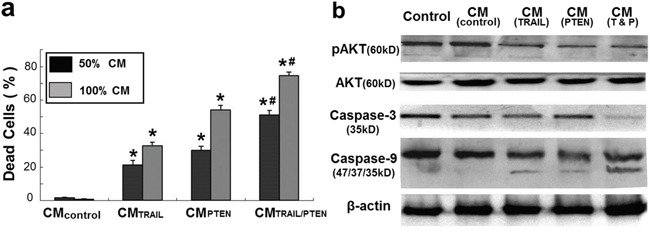
a. Summary of cell viability of indirect co-cultures Mean ± SEM for three independent experiments. *p < 0.05 *vs.* control at the same CM ratio; ^#^ *p* < 0.05 *vs.* CM_PTEN_ at the same CM ratio. **b.** Immunoblotting analysis of apoptosis-related protein expression in DBTRG cells. Cells were harvested at 72 h after indirect co-culture with various CMs. pAKT: phosphorylated AKT.

CM-induced DBTRG cell death was also examined at day 4 with fluorescence microscopy after LIVE/DAED staining. Two CM ratios, 50% and 100%, were used in this part of the study. As shown in Figure 6 and Figure 7a, marked cell death was observed on DBTRG cells incubated with CM_TRAIL_ and CM_PTEN_. It is worthwhile noting that the CM_TRAIL/PTEN_-induced cell death was further increased compared to the treatment with CM_TRAIL_ or CM_PTEN_ (*p* < 0.05) under two tested CM ratios. Figure 7b showed the results of immunoblotting analysis of apoptosis-related proteins in DBTRG cells during indirect co-culture. DBTRG cells expressed similar amount of total AKT after the treatment with various CMs. However, the phosphorylated form of AKT (pAKT, Ser473) was obviously down regulated by the treatment of CM_TRAIL_ and CM_PTEN_ alone or their combination. CM_TRAIL_, CM_PTEN_ and CM_TRAIL/PTEN_-induced procaspase-9 cleavage and caspase-3 activation were also consistent with apoptosis.

### The effects of *TRAIL*- and *PTEN*-engineered MSCs on the tumor growth *in vivo*

**Figure 8:**
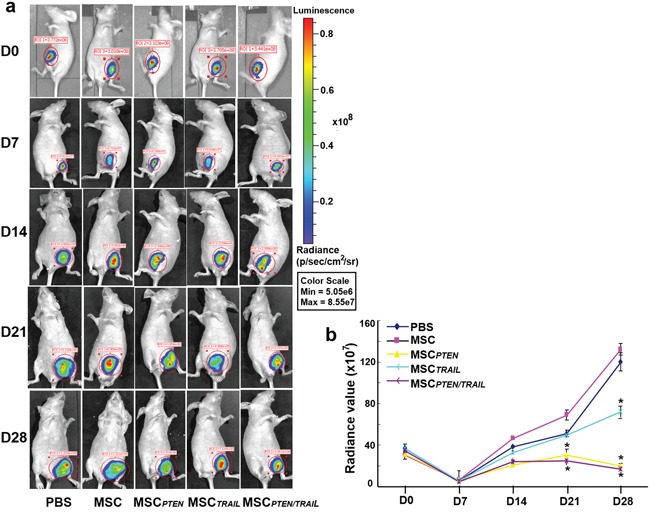
The antitumor effects of *TRAIL*- and *PTEN*-mRNA-engineered MSCs in xenografted animal model Nude mice received xenotransplantation of DBTRG-Luc cells (1×10^7^ cells/each) on day 0 and received initial intravenous injection of various MSCs (0.5×10^6^ cells) on day 7, including native MSCs, MSC*_TRAIL_*, MSC*_PTEN_* and MSC*_TRAIL/PTEN_* (3 mice each group). The mice in control group received injection of the same volume of PBS. The additional cell application was given on days 14 and 21. The whole animal images were taken every week starting from day 0 until the end of the experiment (day 28). Radiance value was used to represent the amount of bioluminescence-producing cells or tumor volume. The representative images were shown in Figure 8a and the time-course of xenografted tumor growth was presented in Figure 8b. Results shown were mean ± SEM (n = 3). ^*^p < 0.05, compared with control.

In order to directly assess the effect of *TRAIL*- and *PTEN*-engineered MSCs on the tumor growth, DBTRG cell-derived xenografted tumors in nude mice were investigated in the present study. As shown in Figure 8, the tumor growth inhibition by antitumor gene-engineered MSCs was exhibited at 7 days (D14) after initial application of the transfected MSCs. Compared with PBS control group, the significant inhibition of the tumor growth was observed on days 21 and 28 for the groups of MSC*_PTEN_* and MSC*_PTEN/TRAIL_* (*P*<0.05). MSC*_TRAIL_*-induced inhibition was only demonstrated on day 28 (*P*<0.05). However, native MSCs seemed to stimulate the growth of the xenografted tumor under the present experimental condition.

## DISCUSSION

Malignant gliomas are the most common and deadly brain tumors with increasing incidence throughout the world [28–30]. The average survival time after surgery and chemoradiotherapy is only 2 to 3 years. In addition to the existence of blood-brain barrier (BBB) and brain-tumor-cell barrier (BTB) in the brain, gliomas' complex cellular composition, diffuse invasiveness and capacity to escape conventional therapies have challenged researchers and hampered progress towards an effective treatment for past three decades [31, 32]. Since the discovery of tumor-directed homing capacity of MSCs, the application of specific antitumor gene-engineered MSCs has attracted great interest in the development of targeted therapy of malignant gliomas [3, 17]. Although the precise molecular mechanism by which MSCs are able to migrate and home into tumor sites is not yet fully clarified, a similar process of leukocytes migrating to inflammation sites has been considered as a paradigm [33]. Tumors can be described as ‘wounds that never heal’, continuously releasing inflammatory mediators including cytokines and chemokines [34]. These biological signals are able to recruit respondent cells including MSCs. The prerequisites for this phenomenon are the production of chemo-attractant molecules from tumor tissue and the expression of corresponding receptors in MSCs [3]. In addition to their tropism feature, the native MSCs possess anticancer properties evidenced by the anticancer effects of MSC-derived soluble factors [15] and MSCs' intrinsic antitumor properties [1]. However, it is worth noting that native MSCs can facilitate tumor growth under certain circumstances [35–37]. The native MSC-induced stimulation of tumor growth is possibly attributed to MSC-derived immunosuppressive factors and the contributions of MSCs to tumor stroma and tumor vascularization [3]. The possible mechanisms and prospective models were summarized in recent reviews [2, 33, 38]. Because tumor tropism properties of MSCs play a pivotal role in MSC-mediated antitumor modality, it is critical to investigate any possible effects of antitumor gene engineering on their tumor-oriented migration as well as their biological features. In the present study, pancreas-derived MSCs were used as the host cells which were characterized by both membrane biomarker and functional differentiation. Human glioma DBTRG cells were used as target cells which were identified by karyotype analysis.

TRAIL is one of few anticancer proteins which selectively causes apoptosis of tumor cells through the activation of death receptors without effects on healthy cells [39]. There are five TRAIL receptors, *i.e.*, TRAIL receptors 1 and 2 (death receptors 4 and 5, DR4 and DR5), TRAIL receptors 3 and 4 (decoy receptors 1 and 2, DcR1 and DcR2), and a soluble receptor, osteoprotegerin (OPG) [40]. DR4 and DR5 death receptors have a death domain in the intracellular region which can recruit DISC (death-inducing signaling complex) upon TRAIL stimulation, and therefore, activate a downstream caspase cascade leading to cell apoptosis. Differing from TRAIL which acts through extracellular receptors, PTEN interacts with multiple intracellular targets [31]. As a phosphatidylinositol phosphatate phosphatase, PTEN directly converts PIP_3_ to PIP_2_ thereby inhibiting PI3K-AKT-mTOR signaling pathway [41]. AKT is a centrally important downstream effector of PIP_3_ which activates AKT through phosphorylation at Ser473 (phosphorylated AKT or pAKT). Since PI3K-AKT-mTOR survival pathway is also an anti-apoptotic pathway, the function of direct opposing PI3K renders PTEN as the central negative regulator of the PI3K-AKT-mTOR pathway in controlling apoptosis [41]. TRAIL and PTEN induce cancer cell apoptosis through extrinsic pathway and intrinsic pathway respectively [42]. The therapeutic range of MSC-mediated modality can be enlarged by using multiple antitumor gene-engineered MSCs, and presumably, a synergistic effect can be obtained through the simultaneous application of multiple antitumor agents.

*TRAIL*-mRNA and *PTEN*-mRNA were constructed and used in this MSC-mediated anticancer study. TRAIL induces tumor cell death specifically through the extrinsic pathway of apoptosis while sparing normal healthy cells. The tumor specificity of TRAIL-induced apoptosis is determined by the death receptor expression in tumor cells [43]. PTEN functions as the central negative regulator of PI3K-AKT-mTOR pathway in controlling apoptosis and plays a critical role in regulating the apoptotic threshold to multiple stimuli, including death ligands and chemotherapeutic agents [41, 44]. The loss of PTEN expression in a wide range of cancer cells (including gliomas) reflects its importance in the maintenance of cancer cell survival [45, 46]. Because MSCs were used as the carrier of these antitumor genes, both TRAIL and PTEN should be expressed inside of MSCs and secreted into the extracellular space. A signal sequence was introduced to the corresponding DNA templates. In consideration of PTEN's intracellular nature, a TAT segment was integrated into the PTEN construct. As demonstrated in Figure 2, the synthesized mRNAs were well expressed in MSCs. The active expression retained at least 4 days with the peak activity on day 2 after transfection. The expression properties are consistent with our previous results obtained with DNA-based vectors in different cell models [19, 47].

In our recent study, the tumor cell-directed migration of MSCs which were engineered with antitumor gene through DNA-based expression vectors was investigated using xCELLigence system in pancreatic cancer cells [20] and using real-time imaging system in glioma DBTRG cells [47] respectively. In order to make the strategy clinically practical, the effects of RNA-based vector transfection on MSCs' migration ability must be validated. As shown in Figure 3, the migration rate of MSCs was not impeded by *TRAIL*-mRNA or *PTEN*-mRNA transfection. Furthermore, MSCs' migration toward DBTRG cells was significantly enhanced by single gene transfection. The underlying mechanisms need to be investigated in future studies. These results provide a solid base for synthesized mRNA vectors to be applied to the MSC-mediated anticancer strategy.

In the present study, the cytotoxic effects of conditioned media from MSC*_TRAIL_* and/or MSC*_PTEN_* on DBTRG cells were examined with luminescence technique, real-time assessment and fluorescence microscopy. Luminescence technique with the aid of IVIS spectrum system is capable for dynamically real-time assessment of cell viability. However, the use of fluorescence microscopy after LIVE/DEAD detects the end-point cell viability, but it is able to provide detailed cellular information. Under the current indirect co-culture condition, DBTRG cells were very sensitive to CM_TRAIL_ and CM_PTEN_ (Figures 4–6). The significant cytotoxicity was observed at very early stage (day 3) and at low CM ratio (25%). It is worth noting that the cytotoxicity was further intensified by CM from MSCs cotransfected with both *TRAIL*-mRNA and *PTEN*-mRNA (MSC*_TRAIL/PTEN_*). Presumably, TRAIL and PTEN acted synergistically inducing cell death on this particular cell type. The western blot analysis of AKT, caspase-3 and caspase-9 was performed to characterize the nature of DBTRG cell death. As shown in Figure 7, the phosphorylated form of AKT (pAKT) was obviously down regulated by the treatment of CM_TRAIL_ and CM_PTEN_ alone or their combination at 72 h, while the expression of total AKT seemed up-regulated with the treatment of CM_PTEN_ and CM_TRAIL/PTEN_. CM_TRAIL_, CM_PTEN_ and CM_TRAIL/PTEN_-induced procaspase-9 cleavage and caspase-3 activation were also detected in this study. The results suggest the apoptotic nature of DBTRG cell death during this indirect co-culture. However, further studies are required to clarify the apoptotic signal pathway under the same experimental condition.

The antitumor effect of *TRAIL*- and *PTEN*-engineered MSCs was directly demonstrated in DBTRG cell-derived xenografted animal models (Figure 8). Compared with MSC*_TRAIL_*, MSC*_PTEN_* showed stronger suppression on tumor growth. However, the synergistic effect of TRAIL and PTEN, which was revealed in the *in vitro* study, was not verified in the *in vivo* animal model. Presumably, the expression levels of death receptors in DBTRG cells might be altered after the formation of xenografted tumor. It is worth noting that the native MSCs showed pro-tumorigenic effect in this study. So, it is critical to make the majority applied MSC cells transfected with antitumor genes, especially when we plan a related clinical trial.

Alongside the advances in genomics and molecular biology, a large number of targeted therapies have been developed in the recent decade. However, current targeted agents exhibit the same frequency and severity of toxicities as traditional cytotoxic agents [48]. A considerable number of cancer patients died from various therapy-related complications. Because of the extraordinary location of glioblastoma, tumor-specific targeted strategy has been eagerly expected. The MSC-mediated therapeutic modality exerts double targeted killing effect to the tumor, mainly due to MSCs' tropism property and engineered antitumor agents. It is able to induce killing effects locally and consistently. This strategy also holds the potential to use patient's own MSCs and to switch tumor attackers corresponding to patient's clinical condition. The present *in vitro* study provides an essential base to use synthesized mRNA in *in vivo* studies and potentially clinical trials. As prospected in our recent review [49], further *in vivo* studies especially combined with the use of intravital biobanks will lead to the development of clinically meaningful antitumor therapy.

In conclusion, *TRAIL*- and *PTEN*-mRNAs were synthesized and studied in an *in vitro* model of MSC-mediated indirect co-culture. The expression of TRAIL and PTEN in transfected MSCs was verified by immunoblotting analysis on cell lysates and conditioned media. The migration ability of MSCs after antitumor gene transfection was demonstrated using transwell co-cultures. A dose-responsible cytotoxicity of DBTRG glioma cells revealed the anticancer effect of MSCs transfected with *TRAIL*- and *PTEN*-mRNAs. The antitumor effect of *TRAIL*- and *PTEN*-mRNA-engineered MSCs was also verified in DBTRG cell-derived xenografted animal models. The results suggested that anticancer gene-bearing mRNAs synthesized *in vitro* are capable of being applied for MSC-mediated anticancer modality. This study provides a solid base for further clinical anticancer studies using synthesized mRNAs.

## MATERIALS AND METHODS

### Cells and culture conditions

MSCs were isolated from human pancreas and *ex vivo* expanded as previously described [19, 20]. They were cultured in MEM with 10% FCS, 2 mM L-glutamine and 1% penicillin-streptomycin solution (all from Invitrogen, Carlsbad, CA, USA) and incubated at 37°C in a humidified, 5% CO_2_ atmosphere. Based on the essential criteria for defining human MSCs established by International Society of Cellular Therapy (ISCT), theses MSCs were verified by both membrane biomarker determination and functional differentiation. The membrane biomarkers were detected with flow cytometry, including CD34, CD44, CD73, CD95 and CD105. Adipogenic and osteogenic differentiations of MSCs were performed using previously described procedures [20]. The MSCs used in this study were limited to passages 3-5. A human glioma cell line (DBTRG) was purchased from American Type Culture Collection (ATCC, through Cedarlane, Burlington, Canada) and used as target cells in the present study. DBTRG cells were maintained as suggested by ATCC and their culture condition was kept consistent with MSCs. In order to track their viability in a timely manner, DBTRG cells were pre-labelled with luciferase by pGL4.51 (Luc/CMV/Neo, Promega) transfection and G418 selection. Chromosome karyotype analysis was performed as previously described [27]. The DBTRG cells used in this study were limited to passages 5-7 counted from the initial culture in our institute.

### Animals

Nude mice (female, 4-6 weeks of age) were purchased from the Model Animal Research Center at Nanjing University (Nanjing, China) and housed in accordance with the National Institutes of Health Guide for the Care and Use of Laboratory Animals. The experimental protocols of the present study were approved by the Animal Care Committee at Hubei University of Medicine (Shiyan, China).

### *In vitro* synthesis of TRAIL- and PTEN-bearing mRNAs

*TRAIL*- and *PTEN*-bearing mRNAs were generated by *in vitro* transcription. The human 5′UTR with Kozak sequence and 3′UTR sequence were synthesized commercially by Integrated DNA Technologies (Coralville, Iowa) and sub-cloned into pcDNA3.3. Plasmid inserts were excised by restriction enzyme digestion and used to template tail PCRs. The templates of human *TRAIL* and *PTEN* were obtained from our previously constructed expression vectors [19, 47]. MEGAscript T7 kit (Ambion) was used to synthesize mRNA. However, m7GpppG was replaced with ARCA cap analog (New England Biolabs) and cytidine and uridine were replaced with 5-methylcytidine triphosphate and pseudouridine triphosphate (TriLink Biotechnologies) respectively. Reactions were incubated 5 h at 37°C followed by DNase treatment. Then, the reactions were treated with Antarctic Phosphatase (New England Biolabs) for 2 h at 37°C to remove residual 5′-triphosphates. The synthesized RNA was purified with Ambion MEGAclear spin columns (Ambion) and quantitated by Nanodrop (Thermo Scientific).

### Transfection of MSCs with synthesized mRNAs

The transfection of *TRAIL*- and *PTEN*-mRNAs into MSCs was carried out with TransIT-mRNA (Mirus). RNA was diluted in Opti-MEM basal media (Gibico) and then, Boost reagent and TransIT-mRNA were added sequentially. After 2 min incubation at room temperature (RT), the RNA-lipid complexes were delivered to culture media in the culture plates. The plates were then returned to the incubator and target gene expression was analyzed 6 and 12 hours later. The MSCs transfected with *TRAIL*-mRNA and *PTEN*-mRNA were defined as MSC*_TRAIL_* and MSC*_PTEN_* respectively.

### Flow cytometry analysis (FACS)

Antibodies for CD34, CD44, CD95, CD105 (phycoerythrin conjugated) and CD34 (allophycocyanin conjugated) and their corresponding isotypes were purchased from eBiosciences (San Diego, USA) and used according to the manufacturer's protocol. Sub-confluent cells were detached with 0.25% trypsin-EDTA and washed with PBS. A total of 1-5 × 10^5^ cells were re-suspended in 200 μl PBS for each reaction, and then 10 μl of primary antibody solution (25 μg/ml) was added to each eppendorf tube and incubated at 4°C for 30 min. The cells were re-suspended with 500 μl PBS for FACS analysis (FACSCalibur, Becton Dickinson, Heidelberg, Germany). For all of the FACS experiments, 15,000 events were recorded and the mean fluorescence intensity of each receptor was assessed on the live cell population. The reference gating location was determined by both isotype IgG control with the aid of Flowjo software purchased from Tree Star (Ashland, OR, USA). For the detection of mRNA transfection efficiency in the MSCs, the GFP-positive cells in *GFP*-mRNA-transfected MSCs were measured using FACS directly.

### Viability assay of tumor cells in indirect co-culture

The cytotoxic effects of TRAIL- and PTEN-engineered MSCs on the proliferation of malignant glioma cells were assessed under indirect co-culture conditions. Luciferase gene transfected DBTRG cells were plated at 5000 cells/well in a BD Falcon 96-well plate in 100 μl of MEM medium on day 0. The media were replaced with conditioned media (CM) that were collected from the cultures of native MSCs (CM_control_), MSC*_TRAIL_* (CM_TRAIL_) and MSC*_PTEN_* (CM_PTEN_) on day 1. Culture volume was 100 μl with five different ratios (CM from MSC*_TRAIL_* or MSC*_PTEN_*/ CM from MSC control), *i.e.* 0%, 25%, 50%, 75%, and 100%. On days 0, 3 and 6, 1 μl of D-luciferin (0.15 mg/ml, Caliper Life Sciences, Hopkinton, MA) was added into each well and the cells were incubated at the same culture condition for additional 10 min. Thereafter, bioluminescence was measured with IVIS Spectrum System (Caliper Life Sciences, Hopkinton, MA). The media were replaced every 36 hrs and the measurements of every group were repeated three times.

The cell viability was also detected by real-time assessment using the xCELLigence real time cell analyzer (RTCA, Roche, USA) as previously described [20, 50]. BDTRG cells were trypsinized and counted using the trypan blue exclusion method and haemacytometer and then were re-suspended in culture medium. Background measurements were taken from the wells by adding 100 μl of the same medium to the E-Plate 16. A volume of 100 μl of cell suspension (6 × 10^3^ cells) was then added to the wells to make a final volume of 200 μl. All cells were allowed to settle at the bottom of the wells at room temperature (RT) for 15 min, and then were incubated at 37°C and 5% CO_2_. The impedance signals were recorded every 5 min for the first 48 h. After 48 h of base line measurement, the culture medium was replaced with CM at different ratios, *i.e.* 0% (control), 25% and 50% respectively. The impedance signals were recorded using the same time intervals until the end of the experiment (up to 120 h). Cell index (CI) value is defined as relative change in measured impedance to background impedance and represents cell status, and is directly proportional to the quantity, size, and attachment forces of the cells.

### Migration assay of MSCs with transwell co-culture system

Cell migration was assayed using the transwell co-culture system (Corning, Inc., Corning, NY). DBTRG cells were placed on plastic surface of the 24-well plates (1 × 10^5^ cells/well). On the following day, the native MSCs and MSC*_TRAIL_* or MSC*_PTEN_* in serum-free medium were seeded on the microporous membrane (8 μm) of the transwell insert respectively. As described by Nakamizo *et al.*[51], MSCs were incubated for 48 hrs at 37°C and 5% CO_2_. The inserts were washed with PBS and the upper surface of the membrane was scraped gently using a cotton swab to remove non-migrated cells. Then, the membranes were fixed with 95% ethanol and stained using 0.1% crystal violet. The average number of migrated cells was assessed by counting five randomly selected microscopic fields at 100x magnification. All experiments were done in triplicate. To detect MSCs' migration status with normal cells, DBTRG glioma cells were replaced with native MSCs on the plastic surface of the 24-well plates.

### Immunoblotting analysis

Immunoblotting analysis was used to detect the cellular expression of TRAIL and PTEN in the MSCs and apoptosis-related proteins (AKT, caspase-3 and caspase-9) in DBTRG cells as previously described [19, 47]. Briefly, MSCs or DBTRG cells were washed with PBS three times and collected with the cell lysis buffer (Beyotime). Cell lysates were incubated on ice for 30 min. Protein concentration was determined using BCA protein assay reagents (Beyotime) according to the manufacturer's protocol. Equal amounts of protein (50 μg/each sample) were loaded on each lane and separated by electrophoresis in 12% SDS ployacrylamide gel and electrotransferred to nitrocellulose membranes. The membrane was put in blocking buffer for 1 hr at RT followed by overnight incubation at 4°C with appropriate primary antibodies (R&D Systems, Minneapolis, USA). The blots were rinsed with TBST three times and incubated with horseradish peroxides-conjugated secondary antibody (1:1000) for 60 min and detected by chemiluminescence using ECL Hyperfilm. The TRAIL and PTEN in the conditioned media were detected after the CM samples were filtered through 0.22 μm membrane and equally concentrated using 10,000 MWCO (cat # 42406; Millipore, Millerica, MA, USA).

### Fluorescence microscopy

The cell viability was also detected using a LIVE/DEAD Viability/Cytotoxicity Assay Kit (Invitrogen) as per the manufacturer's instruction with a slight modification. Briefly, a total of 1×10^5^ DBTRG cells were plated onto 24-well plates in 500 μl of MEM medium on day 0. The media were replaced with 50 or 100% conditioned media on day 1. On day 4, the cultures were washed twice with PBS. Freshly prepared working solution (250 μl per well on 24-well plates, containing 1μM calcein AM and 2 μM EthD-1) was then added directly to the cultures and incubated at room temperature for 10 min in the dark. The images were taken using a fluorescence microscope (IX71; Olympus) and the related analysis was performed through ImageJ (provided online by the National Institute of Health of USA).

### Xenograft test

Since IVIS Spectrum System is preferentially sensitive to bioluminescence, luciferase gene-transfected DBTRG cells (DBTRG-Luc cells) were used for the xenograft test. The cells were harvested when they reached to logarithmic phase of growth. Then, 1×10^7^ of DBTRG-Luc cells were subcutaneously injected into flanks of nude mice. At day 7 after xenotransplantation, the xenografted mice were randomly divided into five groups (3 mice each group) according to the different treatments received, *i.e.* PBS (100 μl), native MSCs, MSC*_PTEN_*, MSC*_TRAIL_* and MSC*_PTEN/TRAIL_* (0.5×10^6^ cells in 100 μl PBS). The MSC cells or PBS were intravenously injected through the tail veins on days 7, 14 and 21 respectively. The xenografted mice were subject to a series of imaging detections under anesthesia. Anesthesia was performed using intraperitoneal injection of pentobarbital (90 mg/kg; Shanghai Chemical Reagent, Shanghai, China). The sizes of grafted tumors were measured every 7 days using a small animal image system (IVIS, Caliper Life Sciences, Hopkinton, MA, USA) until the end of the experiment (day 28). Each test was conducted 10 min after anesthetized mouse received 200μl D-luciferin solution (0.15 mg/ml, Caliper Life Sciences) intraperitoneal injection. The growth rate and size of tumors were analyzed using the radiance value which is proportional to the number of bioluminescence-producing cells [52].

### Statistical analysis

Numerical data were expressed as mean ± standard error. Statistical differences between the means for the different groups were evaluated with Prism 4.0 (GraphPad software, La Jolla, CA, USA) using the Student's *t*-test with the level of significance at *p*<0.05.
